# Vacuolating cytotoxin A (VacA), a key toxin for *Helicobacter pylori* pathogenesis

**DOI:** 10.3389/fcimb.2012.00092

**Published:** 2012-07-12

**Authors:** Samuel L. Palframan, Terry Kwok, Kipros Gabriel

**Affiliations:** ^1^Host Pathogens Molecular Biology Group, Department of Biochemistry and Molecular Biology, Monash University, ClaytonVIC, Australia; ^2^Department of Microbiology, Monash University, ClaytonVIC, Australia

**Keywords:** *Helicobacter pylori*, VacA

## Abstract

More than 50% of the world's population is infected with *Helicobacter pylori* (*H. pylori*). Chronic infection with this Gram-negative pathogen is associated with the development of peptic ulcers and is linked to an increased risk of gastric cancer. *H. pylori* secretes many proteinaceous factors that are important for initial colonization and subsequent persistence in the host stomach. One of the major protein toxins secreted by *H. pylori* is the Vacuolating cytotoxin A (VacA). After secretion from the bacteria via a type V autotransport secretion system, the 88 kDa VacA toxin (comprised of the p33 and p55 subunits) binds to host cells and is internalized, causing severe “vacuolation” characterized by the accumulation of large vesicles that possess hallmarks of both late endosomes and early lysosomes. The development of “vacuoles” has been attributed to the formation of VacA anion-selective channels in membranes. Apart from its vacuolating effects, it has recently become clear that VacA also directly affects mitochondrial function. Earlier studies suggested that the p33 subunit, but not the p55 subunit of VacA, could enter mitochondria to modulate organelle function. This raised the possibility that a mechanism separate from pore formation may be responsible for the effects of VacA on mitochondria, as crystallography studies and structural modeling predict that both subunits are required for a physiologically stable pore. It has also been suggested that the mitochondrial effects observed are due to indirect effects on pro-apoptotic proteins and direct effects on mitochondrial morphology-related processes. Other studies have shown that both the p55 and p33 subunits can indeed be efficiently imported into mammalian-derived mitochondria raising the possibility that they could re-assemble to form a pore. Our review summarizes and consolidates the recent advances in VacA toxin research, with focus on the outstanding controversies in the field and the key remaining questions that need to be addressed.

## Introduction

*Helicobacter pylori* (*H. pylori*) is a Gram-negative bacterium that colonizes the human stomach. Approximately half of the human population worldwide is infected with *H. pylori*. Infection is spread through human contact primarily via the gastric-oral route and is often acquired in early childhood (Marshall, 1991; Blaser et al., 2004; Amieva and El-Omar, 2008). If untreated, *H. pylori* infection often persists for life, highlighting the remarkable adaptation of this pathogen to the human stomach. Chronic superficial gastritis is a hallmark of persistent *H. pylori* infection and is usually asymptomatic in most infected individuals. However, approximately 10–15% of the infected population develop severe gastric disorders including peptic ulcers, gastric lymphoma, mucosa-associated lymphoid tissue (MALT) lymphoma and gastric adenocarcinoma (Marshall and Warren, 1984; Marshall, 1991; Blaser et al., 2004; Houghton and Wang, 2005). Gastric adenocarcinoma is the second leading cause of cancer-related deaths worldwide, following only lung cancer (Ferlay et al., 2008). Intriguingly, isolated studies have suggested that *H. pylori* might also be a causative agent of non-gastric diseases such as vascular disease, chronic liver disease and idiopathic thrombocytopenic purpura (Pellicano et al., 2009).

One of the most extensively studied toxins produced by *H. pylori* is the Vacuolating cytotoxin A (VacA). Infection with *H. pylori* strains containing the toxigenic allelic *s1* form of VacA is associated with an increased risk of peptic ulceration and gastric cancer (Atherton et al., 1995; Gerhard et al., 1999; Miehlke et al., 2000, 2001; Louw et al., 2001). VacA was named with reference to its ability to cause “vacuole”-like membrane vesicles in the cytoplasm of gastric cells (de Bernard et al., 1997), but their roles in *H. pylori* pathogenesis remain unclear. In addition to the induction of vacuolation, VacA exerts a variety of other effects on target cells, including disruption of mitochondrial functions, stimulation of apoptosis and blockade of T-cell proliferation (Cover and Blanke, 2005). VacA is also important for colonization of *H. pylori in vivo* (Salama et al., 2001). Given the fascinating multi-functionality of VacA and its association with an augmented gastric cancer risk, understanding the biochemical properties of this versatile toxin and how it contributes to *H. pylori* pathogenesis are areas of active and intensive research. In this review we summarize key recent findings, controversies and unanswered questions relating to VacA biology.

## VacA—A multi-functional toxin

**Figure 1:**
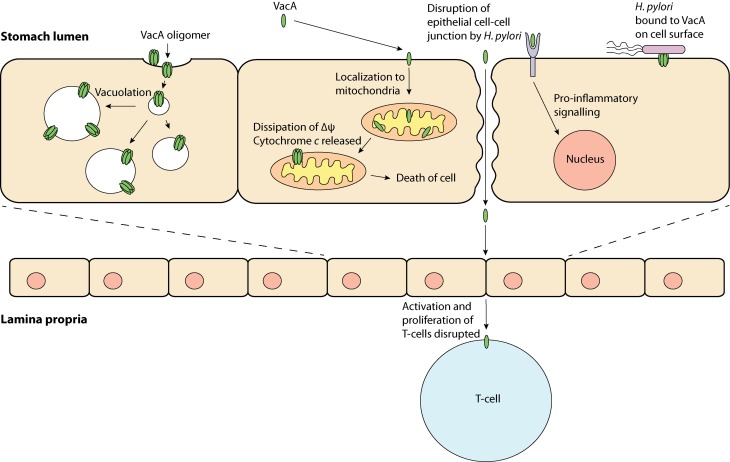
**VacA, a multi-functional toxin—VacA may produce “vacuoles,” which have traits of late endosomes and early lysosomes; be taken up by the cell and localize to the mitochondria, which may result in apoptosis; bind to a protein on the cell membrane and induce inflammation and; obstruct T-cell activation and proliferation**.

Research in the past few decades has revealed that VacA has a variety of effects on host cells and has been termed a “multi-functional toxin.” Vacuolation is perhaps the most distinct effect of VacA. This pronounced accumulation of internal membranous vesicles (Figure 1) occurs following VacA internalization by the host cell. VacA is hypothesized to create anion-selective channels in the membranes of these vesicles that possess hallmarks of both late endosomes and early lysosomes (Papini et al., 1994). A current model for vacuolation suggests that these anion-selective channels facilitate the transport of chloride ions, resulting in an increase in intra-lumenal chloride concentrations (Cover and Blanke, 2005). Ultimately, membrane-permeable weak bases diffuse into these endocytic compartments resulting in osmotic swelling and vacuolation (Cover and Blanke, 2005). A hydrophobic region on the N-terminal domain of VacA (amino acids 6–27) has been shown to be required for “vacuolation” (Vinion-Dubiel et al., 1999). Interestingly, removing this hydrophobic region of VacA not only inhibits vacuolation but also prevents VacA from stably integrating into the inner-mitochondrial membrane (Vinion-Dubiel et al., 1999; Foo et al., 2010). The physiological significance of “vacuolation” during *H. pylori* infection is not clear, although it is plausible that “vacuolation” could disrupt protein trafficking pathways to and from the plasma membrane, hence exerting far-reaching effects on host cell functions.

In addition to its role in the induction of vacuolation, VacA has been shown to localize to mitochondria where its effects may be responsible for triggering the apoptotic cascade (Kimura et al., 1999; Galmiche et al., 2000; Foo et al., 2010). Typically, during apoptosis cytochrome *c* is released from the mitochondrial inter-membrane space into the cytoplasm via an unknown mechanism; downstream executioner caspases are then activated, which ultimately result in cell death. In line with the proposed pore-forming capabilities of VacA, it has been hypothesized that VacA can form membrane-embedded pores at the inner-mitochondrial membrane resulting in the dissipation of the mitochondrial electrochemical membrane potential (Δψ) (Willhite and Blanke, 2004). Although the VacA-mediated reduction in Δψ has been suggested to accompany the release of cytochrome *c* (Kimura et al., 1999; Galmiche et al., 2000), the exact apoptotic role of VacA remains unclear, as a drop in the Δψ alone is not expected to cause apoptosis (Yamasaki et al., 2006). Despite this observation, previous studies have shown that inhibition of VacA membrane channel formation inhibits the release of cytochrome *c*, suggesting that channel formation is critical to the apoptotic potential of VacA (Willhite et al., 2003; Willhite and Blanke, 2004).

## Polymorphism, biogenesis, and structure of VacA

**Figure 2:**
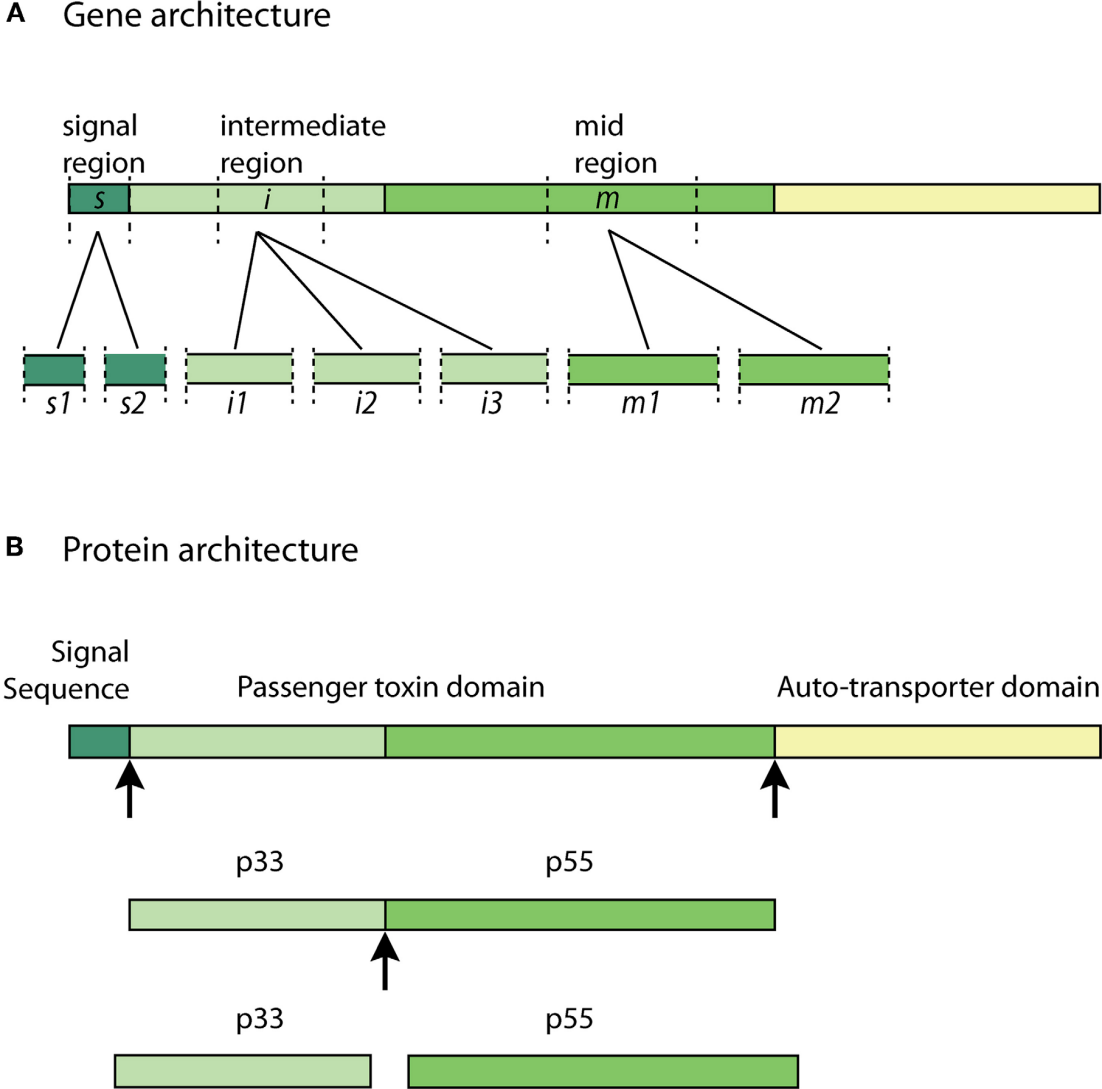
**VacA allelic diversity and structure—(A) significant allelic diversity exists in three regions of the VacA gene: the signal region (*s1* and *s2*), the intermediate region (*i1*, *i2*, and *i3*) and the mid-region (*m1* and *m2*); (B) the Signal Sequence allows for the passage of the pro-toxin across the inner-bacterial membrane. The passenger toxin domain consists of the N-terminal VacA fragment (p33) and the C-terminal VacA fragment (p55). The auto-transporter domain allows the toxin to translocate across the outer-bacterial membrane by forming a β-barrel. The p33 and p55 fragments may be cleaved from the β-barrel domain at some point during transit to, or in, the extracellular milieu to form the mature virulent subunits. Arrows indicate cleavage sites**.

All identified *H. pylori* strains possess the VacA gene. However, there is significant sequence diversity in *vacA* genes across the many *H. pylori* isolate strains (Atherton et al., 1995; Van Doorn et al., 1999; Rhead et al., 2007). Polymorphism in the *vacA* gene sequence has been identified in three variable regions: the signal sequence region (*s*-region), mid-region (*m*-region) and the recently identified intermediate-region (*i*-region) (Figure 2A) (Atherton et al., 1995; Rhead et al., 2007).

The two types of allelic variations in the *s*-region and *m*-region are classified as *s1* or *s2* and *m1* or *m2*, respectively (Atherton et al., 1995). The *s2* type encodes a VacA protein with an additional N-terminal hydrophilic amino acid segment, which the *s1* type lacks. The presence of this extra segment prevents the *s2* type toxin from inducing vacuolation (McClain et al., 2001). The *m1* and *m2* genotypes differ with respect to an encoded stretch of 148 amino acids, which ultimately affects the specificity to various cellular receptors based on the observed differences in VacA activity (Ji et al., 2000). It has been shown that *H. pylori* with *s1/m1* and *s1/m2* VacA cause more severe chronic-inflammation when compared to the other genotypes. The highest level of virulence is associated with the *s1/m1* allele combination, which is also associated with the highest increased risk of gastric cancer (Atherton et al., 1995; Gerhard et al., 1999; Miehlke et al., 2000, 2001; Louw et al., 2001). Recent studies investigating the *i*-region have revealed that the *i1* allele strongly correlates with the production of CagA (a virulence factor encoded by the cytotoxin-associated gene A) and the presence of the *s1* type allele in various *H. pylori* strains isolated from several countries (Chung et al., 2010). This association could suggest that the intermediate region plays a role in the more severe outcomes of chronic *H. pylori* infection (Chung et al., 2010).

The VacA gene encodes a 140 kDa pro-toxin. The pro-toxin consists of a signal sequence, a passenger domain and an auto-transporter domain; the latter of which functions as a type V secretion system. The passenger domain, containing the p33 and p55 subunits, are later processed and cleaved from the auto-transporter domain at some point during transit to, or in, the extracellular milieu to form the mature virulent 88 kDa VacA toxin (Telford et al., 1994; Lupetti et al., 1996; Isomoto et al., 2010). The pro-toxin and cleavage sites are illustrated in Figure 2B. The N-terminal fragment with a molecular weight of approximately 33 kDa is termed “p33” (also known as p34 and p35) (Cover and Blanke, 2005). The C-terminal fragment with an approximate molecular weight of 55 kDa is termed “p55” (also known as p58) (Cover and Blanke, 2005; Isomoto et al., 2010).

The cellular role of each VacA subunit is still not fully understood. The pore-forming activity of VacA was considered to be p33 dependent, whilst cell binding was initially suggested to be dependent on the p55 domain (Reyrat et al., 1999; McClain et al., 2003). It has since been demonstrated that p33 is also involved in cell binding, and recently it was shown that p55 is in fact essential for vacuolation and membrane depolarization, suggesting p55 is involved in the formation of anionic membrane channels (Torres et al., 2005; Ivie et al., 2008; Gonzalez-Rivera et al., 2010). Furthermore, the crystal structure of p55 has been solved, it consists of a series of parallel β-strands with a carboxy-terminal globular domain (Gangwer et al., 2007). Modeling the predicted structure of p33 with the solved crystal structure of p55, gives rise to a model of the molecular architecture of the assembled pore, which stipulates that a stable anionic membrane channel can only be formed when both subunits are present (Gangwer et al., 2007).

## VacA entry into host cells

The binding of VacA to a target cell is a critical event in its toxicity. As VacA affects multiple cell types including epithelial cells and T-cells, it is likely that a range of host cell receptors or even other surface factors are involved in cell binding (Yahiro et al., 1999, 2003; Sewald et al., 2008). Unsurprisingly, the receptors utilized by VacA when binding to T-lymphocytes differ to those when binding to gastric epithelial cells. Recently it was revealed that the β2 integrin subunit of lymphocyte function-associated antigen-1 (LFA-1), CD18, is in fact a specific VacA receptor in T-cells (Sewald et al., 2008). In contrast, the receptors implicated in the binding of VacA to gastric epithelial cells include the epidermal growth factor (EGF) receptor, heparin sulphate, glycosphingolipids, receptor protein tyrosine phosphatase alpha (RPTPα), receptor protein tyrosine phosphatase beta (RPTPβ) and sphingomyelin among others (Seto et al., 1998; Yahiro et al., 1999, 2003; Utt et al., 2001; Roche et al., 2007; Gupta et al., 2008).

RPTPβ was discovered as a possible receptor for VacA when the presence of intracellular vacuoles was linked to the enhanced binding of the toxin to a 250 kDa glycoprotein found on the surface of AZ-521 cells (a malignant gastric tumor cell line). The glycoprotein was later identified as RPTPβ (Yahiro et al., 1999). Several years later, VacA was shown to cause vacuolation in G401 cells (a kidney tumor cell line) (Yahiro et al., 2003). Vacuolation occurred in these cells despite the lack of RPTPβ on the cell surface. To further investigate this, VacA was co-immunoprecipitated after incubation with G401 cells. A glycoprotein of 140 kDa (p140) was identified as a receptor for VacA and was found to be RPTPα (Yahiro et al., 1999, 2003).

Although RPTPα remains a possible VacA cellular receptor, a recent study has cast doubt on the classification of RPTPβ as an essential epithelial receptor for VacA, as it was discovered that vacuolation occurred in HeLa cells lacking RPTPβ (Skibinski et al., 2006).

Interestingly, specific binding to individual cell types has been attributed to the *m1* and *m2* alleles of the VacA gene. VacA of the *s1*/*m1* type successfully bound to target HeLa cells and induced vacuolation, whereas the *s1*/*m2* VacA affected rabbit-kidney (RK13) cells and primary epithelial cells but not HeLa cells (Pagliaccia et al., 1998; Ji et al., 2000). RPTPα and RPTPβ are both recognized by *s1*/*m2* VacA (De Guzman et al., 2005). Because the *m*-region alleles are located on the p55 domain, these findings support the hypothesis that p55 is involved in cell binding.

## Targeting of VacA to mitochondria and the apoptotic pathways

Mitochondria are the site of aerobic respiration and are essential to all human cells. They have many functions, including the production of adenosine triphosphate (ATP), fatty acid synthesis and iron sulphur cluster biogenesis. Mitochondria are also crucial to the intrinsic apoptotic pathway through which a cell is instructed to suicide. A series of cellular events culminates in the release of cytochrome *c* from mitochondria into the cytosol; this in turn activates a series of pro-caspases resulting in cell death (Scheffler, 2001).

**Figure 3:**
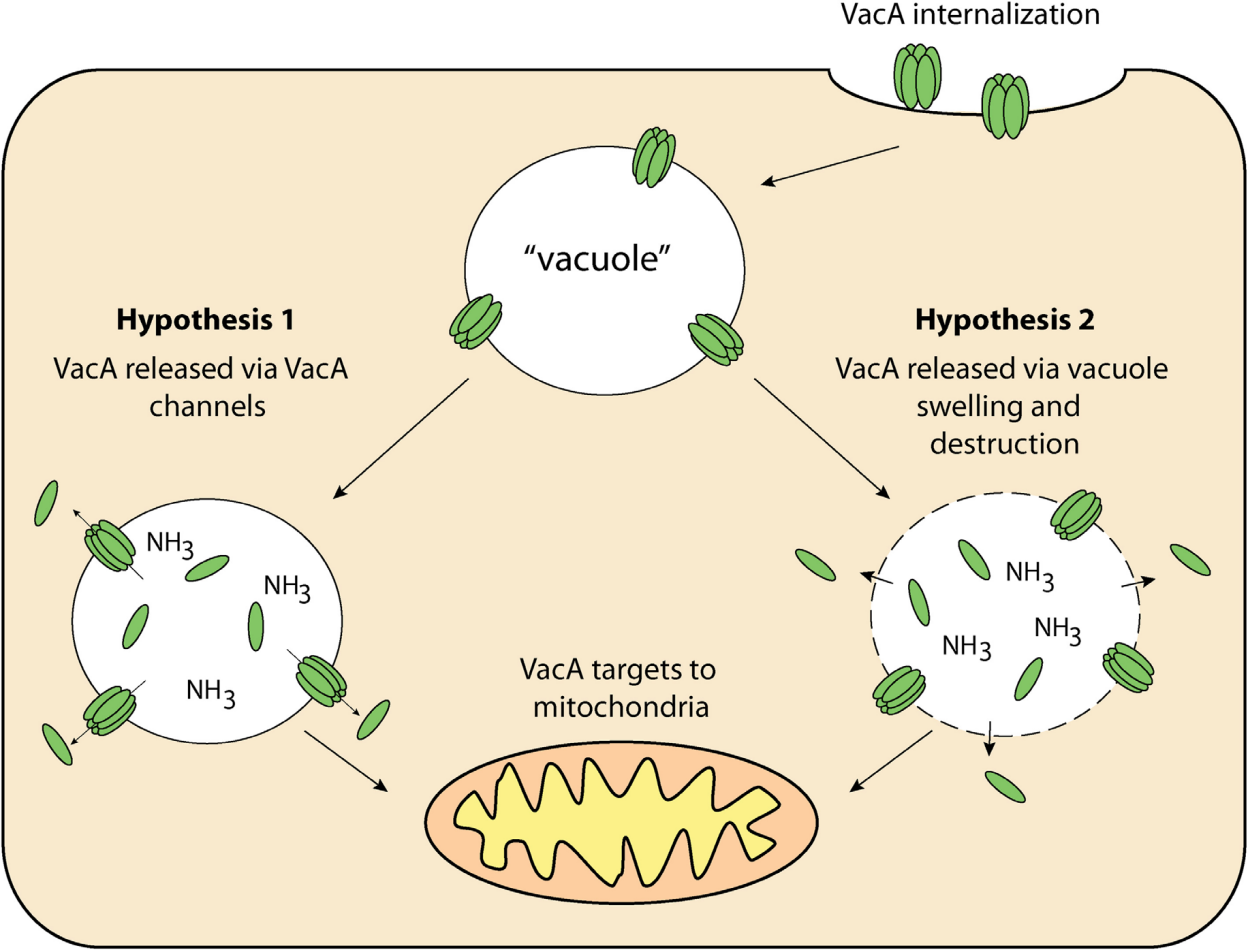
**VacA delivery to mitochondria—once internalized by the host cell, VacA may then be released from “vacuoles” for targeting to the mitochondria via 1: VacA anionic channels within “vacuole” membranes and/or; 2: Compromised “vacuole” membranes resulting from osmotic swelling and eventual destruction**.

The targeting pathway involved in the trafficking of VacA to mitochondria is not fully understood. VacA must first reach the cytosol after having traversed the plasma membrane. After this has occurred, VacA is trafficked to the mitochondria and then translocated across the outer mitochondrial membrane (Willhite and Blanke, 2004; Calore et al., 2010). In order for proteins to cross the outer mitochondrial membrane, they must pass through the Translocase of the Outer Mitochondrial membrane (TOM) (Chacinska et al., 2009). This protein-conducting channel has a diameter that is insufficient for translocation of folded proteins meaning that VacA must be unfolded before or during translocation to thread through the channel and enter mitochondria (Schwartz and Matouschek, 1999; Gabriel and Pfanner, 2007). That aside, recent studies have outlined the importance of the N-terminal domain of VacA in mitochondrial trafficking. It was shown that VacA from wild-type *H. pylori* culture supernatant accumulated in both endosomal and mitochondrial subcellular fractions of mouse embryonic fibroblast (MEF) cells (Calore et al., 2010). In contrast, MEF cells incubated with mutant *H. pylori* culture supernatant, which contained a single-amino acid substitution in the N-terminal region of VacA, prevented the toxin from reaching the mitochondria, and instead remained within the endosomal subcellular fractions. Interestingly, this N-terminal region is also essential for VacA membrane channel formation (Calore et al., 2010), suggesting that channel formation may be important for VacA release from the endosomal compartments to the cytosol from where it can target to mitochondria. This hypothesis is summarized in Figure 3. Although there are many uncertainties surrounding how VacA is delivered to the cytosol and subsequently targeted to mitochondria, it is clear that VacA can indeed ultimately translocate into the organelle. The manner in which this is achieved is still unknown (Galmiche et al., 2000; Willhite and Blanke, 2004; Yamasaki et al., 2006; Foo et al., 2010).

## Current models on VacA-mediated apoptosis

The mechanism responsible for VacA-induced apoptosis is another area of considerable controversy. It has been suggested that the p33 subunit of VacA is targeted to mitochondria and capable of initiating apoptosis alone, independent of p55 (Galmiche et al., 2000; Domanska et al., 2010). Enhanced green fluorescent protein (eGFP) was expressed as a fusion to the N- or C-terminus of p33 or p55. These chimeric proteins were expressed intracellularly within HEp-2 cells and could be tracked for localization using immunofluorescence microscopy. All tagged forms of p33 were localized to mitochondria, whereas GFP-tagged p55 (N- or C-terminally tagged) was not (Galmiche et al., 2000). It is possible the GFP moiety disrupted protein trafficking of the p55 subunit as GFP has been reported to affect the localization of some proteins, particularly those localized to the inter-membrane space (Gabriel et al., 2007). An alternative to the use of large epitope tags would be to raise antibodies against VacA, p33 and p55 that could be used for immunofluorescence experiments with untagged proteins. This would circumvent any changes to protein trafficking pathways caused by the large epitope tags and under such conditions p55 may also be found to localize to mitochondria.

It is important to note that in all previous cell culture studies where the endogenously expressed VacA was used for apoptotic assays, cell death was not measured for at least 24 h from the time of exposure, leaving open the possibility of other secondary effects. This has been compounded further by the use of the Cytomegalovirus (CMV) promoter for expression, which strongly enhances transcription of the gene of interest (Ramanathan et al., 2005). Under these expression conditions there would be an unnaturally high level of VacA in cells, many thousand times more than what would be expected during infection. In addition, the high expression of inter-membrane space accumulating proteins does indeed kill cells through indirect effects (Kozjak-Pavlovic et al., 2010). This raises the possibility that the p33 and p88 (fused p33 and p55) toxin GFP fusions used in studies such as the Galmiche study in 2000, may not be directly responsible for apoptosis *per se* (Galmiche et al., 2000).

More recently, it was again suggested that only the p33 subunit of VacA is targeted to the mitochondria and is essential for toxicity (Domanska et al., 2010). It was hypothesized that a signal sequence of 32 uncharged amino acid residues found on the N-terminus of the p33 subunit, targets it to the mitochondria (Domanska et al., 2010). This interesting observation, however, remains to be reconciled with the previous findings that p33 tagged with GFP at its N-terminus, was still targeted to the mitochondria (Galmiche et al., 2000), as fusing GFP to the N-terminus of p33 would ordinarily mask an N-terminal signal. Furthermore, it was also reported that the p33 subunit forms a pore without p55, but only in the presence of biochemical cross-linkers (Domanska et al., 2010). In contrast, other publications suggest that both p33 and p55 are required for pore formation and vacuolation (Torres et al., 2005; Calore et al., 2010; Foo et al., 2010), and that both subunits could localize to the mitochondrial inter-membrane space. The latter is on the back of evidence that both p33 and p55 can be efficiently imported into mitochondria using an *in vitro* import system (Foo et al., 2010). Residues 6–27 in p33 and internal targeting signals in the regions close to the N-terminus and C-terminus of p55 were shown to be important for stable integration of the subunits into the inner-mitochondrial membrane (Foo et al., 2010). Moreover, import of p55 alone did not result in integral association of p55 with the mitochondrial membranes (Foo et al., 2010). It is thus plausible for both subunits to interact with the inner-mitochondrial membrane and insert, forming the characteristic “star-shaped” pore described in previous studies (El-Bez et al., 2005; Gangwer et al., 2007; Foo et al., 2010). It has been proposed that this “star-shaped” pore consists of six VacA monomers (hexamer) and functions as an anion-selective channel (El-Bez et al., 2005), facilitating the dissipation of the Δψ; a feature linked to the induction of apoptosis (Willhite and Blanke, 2004). Disruption of the Δψ occurs prior to mitochondrial outer-membrane permeabilization (MOMP) (Willhite and Blanke, 2004), the latter of which could serve to function as a mechanism for cytochrome *c* release from mitochondria.

Indeed, the ability of VacA to form membrane channels has been shown to be essential to VacA-induced apoptosis. Mutant forms of VacA that lack the capacity to form membranous channels fail to induce apoptosis in CcGFP-HeLa cells (a cell line expressing a cytochrome *c* and GFP fusion protein). This outcome was reproduced when the same cell line intoxicated with *s1/m1* VacA was incubated with a channel inhibitor (NPPB) (Willhite et al., 2003; Willhite and Blanke, 2004). The finding was confirmed several years later when two isogenic mutant *H. pylori* strains, containing single point mutations in the N-terminal region of VacA which abolish the toxin's ability to form membranous pores, failed to induce apoptosis in MEF cells (Calore et al., 2010). From these data it appears that pore formation is in fact essential for VacA-induced apoptosis via the mitochondrial-dependent pathway.

Recently the story has taken a further twist as it has been suggested that VacA acts externally to mitochondria to induce apoptosis through indirect mechanisms. VacA (more specifically p55) was shown to up-regulate the expression of Bax (a multi-domain pro-apoptotic protein) and VDAC1 (an endogenous outer-mitochondrial membrane channel) resulting in the VacA-induced MOMP and subsequent release of cytochrome *c* (Lan et al., 2010). Furthermore, down-regulation of the anti-apoptotic protein Bcl-2 was also observed, strengthening the case for VacA-induced apoptosis via a mechanism that is not initiated at mitochondria even though mitochondria are involved in later phases (Lan et al., 2010). In a study conducted in the same year, Bax and Bak (a membrane-bound multi-domain pro-apoptotic protein) were found to be essential for *Helicobacter*-induced apoptosis. Double knockout MEF cells deficient for both Bax and Bak were resistant to apoptosis when exposed to *H. pylori* culture supernatant, whereas wild-type MEF cells underwent apoptosis as expected (Calore et al., 2010).

A recent study has revealed that VacA can engage the mitochondrial fission machinery, causing mitochondrial morphology changes that are implicated in VacA-induced cell death (Jain et al., 2011). It was shown that VacA recruits and activates dynamin-related protein 1 (Drp1), a regulator of mitochondrial fission within cells. In AZ-521, AGS and polarized MDCK cells infected with *H. pylori*, mitochondrial networks transition from filamentous networks of interconnected strands to shorter punctiform organelles within 8 h of infection. In cells infected with a *H. pylori* VacA knockout strain, no mitochondrial fragmentation was visible. Critically, it was shown that the inhibition of Drp1-induced mitochondrial fission prevented the activation of Bax, MOMP, and consequently cell death (Jain et al., 2011).

Whilst the intricate balance between apoptosis, fission, fusion, and autophagy is a topic of intense study, VacA has recently been found to trigger autophagy in AGS cells (a gastric adenocarcinoma cell line) (Terebiznik et al., 2009). Autophagy induction by VacA appears to require the toxin pore-forming activity, as culture supernatant of *H. pylori* strains incapable of pore formation did not induce autophagy (Terebiznik et al., 2009). It would be of interest to examine in further detail the causal relationship between pore formation, autophagy induction, and apoptosis induction by VacA.

It is clear that there are still many unanswered questions surrounding the mechanism by which VacA is able to induce apoptosis in gastric epithelial cells, and there is also much debate regarding which of the two subunits (p33 and/or p55) is responsible. Improving the assays used for measuring VacA effects on host cells is required. This may be in the form of introducing a set of vectors that allow for a more tightly regulated and controllable expression of VacA and the expression of untagged versions of the toxin subunits.

## VacA and CagA: the yin and yang of *H. pylori*-induced cytotoxicity

Another key *H. pylori* virulence factor is the gene product of the CagA. Interestingly, recent findings suggest that the effects elicited by CagA on the host cell can counteract those triggered by VacA, and *vice versa*, pointing to yet another level of complexity in the mode of action of VacA. CagA is delivered into the host cell by *H. pylori* via a type IV secretion system (Segal et al., 1999; Asahi et al., 2000; Backert et al., 2000; Odenbreit et al., 2000; Stein et al., 2000). Once inside the host cell, CagA is phosphorylated by the host tyrosine kinase Src (Selbach et al., 2002). Phosphorylated CagA dysregulates actin cytoskeletal rearrangement in the host cell, triggering the so-called hummingbird phenotype, which is typified by an elongated morphology of the host cell that resembles the long thin beak of a hummingbird (Segal et al., 1999).

Functional antagonism between VacA and CagA became apparent when the effects of these two proteins on the activity of the host transcription factor nuclear factor of T-activated cells (NFAT) were examined (Yokoyama et al., 2005). Purified VacA (at 2.5 μg/ml) counteracts the stimulatory effect of CagA on the transcriptional activity of NFAT in cultured gastric epithelial cells (Yokoyama et al., 2005). Nuclear translocation of NFAT was also abolished by the VacA treatment (Yokoyama et al., 2005). Later, Argent and co-workers studied the *H. pylori* strains 60190 and 84–183 and found that VacA-mediated vacuolation attenuated CagA-induced hummingbird phenotype, and *vice versa* (Argent et al., 2008). Tegtmeyer and co-workers confirmed these effects using the *H. pylori* strains P310 and P277, and took a step further to demonstrate that VacA not only inhibited hummingbird phenotype but also increased internalization of epidermal growth factor receptor (EGFR) and inhibited the activities of EGFR and the mitogen-activated protein kinase Erk1/2 (Tegtmeyer et al., 2009). Interestingly, Bafilomycin A1, an inhibitor of V-ATPase and VacA-mediated vacuolation, did not restore hummingbird phenotype in P310-infected cells, suggesting that the inhibitory effect of VacA is unlikely to be due to vacuolation *per se* (Tegtmeyer et al., 2009). These findings suggest that VacA counteracts CagA-mediated hummingbird phenotype by interfering with EGFR- and Erk1/2-mediated signaling pathways. In further support of a functional antagonism between VacA and CagA, a GFP fusion of the 38 kDa C-terminal domain of CagA was shown to inhibit the apoptotic and vacuolating effects of VacA, possibly via inhibition of the activity of Src kinase family (Oldani et al., 2009). This C-terminal domain construct of CagA also blocks co-localization of VacA with LAMP1, possibly inhibiting the trafficking of VacA to late endosomes (Oldani et al., 2009).

Despite the lack of a genetic linkage between the *cagA* and *vacA* genes, most *H. pylori* strains that harbor the *cagA* gene carry the more toxigenic *s1* form of *vacA* allele whereas *cagA*-negative strains usually possess the non-toxic *s2* form of *vacA* (Atherton et al., 1995). The reason for this linkage had been enigmatic. These recent findings have now offered plausible explanations for a functional linkage between CagA and VacA at the level of host cell signal transduction. Such antagonism between CagA and VacA could enable *H. pylori* to take command of host cell responses without causing gross cellular damage. However, the fact that some *H. pylori* isolates are fully capable of both CagA-mediated cellular responses and VacA-induced vacuolation suggests that the net effect of VacA and CagA might vary between strains and could possibly be influenced by the amount of VacA produced or by as yet unidentified factors. Further understanding of the molecular basis of VacA- and CagA-mediated signaling in the host cell is likely to provide crucial insights into the physiological significance of the crosstalk between these two important and intriguing *H. pylori* cytotoxins.

## Concluding remarks

VacA has been implicated as a key *H. pylori* virulence factor and has been shown to exert many cellular effects. Although this multi-functional toxin has been researched extensively, considerable controversy surrounds the fundamental aspects of VacA action and biology. The subunit and cellular receptor responsible for VacA binding and subsequent internalization remain elusive and there is conjecture as to whether a certain receptor identified (RPTPβ) is essential. In addition, little is known about the trafficking of VacA to mitochondria, where VacA is said to induce apoptosis via an unknown mechanism; the most highly disputed area of VacA research. Although many believe both p33 and p55 are required to form an anion-selective channel within the inner-mitochondrial membrane, others postulate that p33 alone is sufficient and capable of forming stable membrane channels independent of p55. Adding further uncertainty, recent evidence has revealed that p55 may indirectly induce apoptosis by indirectly up-regulating pro-apoptotic factors. Delineating the subunit/s responsible will provide crucial insights into the mechanisms at work and could serve as a basis for future studies.

### Conflict of interest statement

The authors declare that the research was conducted in the absence of any commercial or financial relationships that could be construed as a potential conflict of interest.
